# Rapid Antigen-Capture Assay To Detect West Nile Virus in Dead Corvids

**DOI:** 10.3201/eid0911.030318

**Published:** 2003-11

**Authors:** Robbin Lindsay, Ian Barker, Gopi Nayar, Michael Drebot, Sharon Calvin, Cherie Scammell, Cheryl Sachvie, Tracy Scammell La Fleur, Antonia Dibernardo, Maya Andonova, Harvey Artsob

**Affiliations:** * National Microbiology Laboratory, Health Canada, Winnipeg, Manitoba, Canada; † University of Guelph, Guelph, Ontario, Canada; ‡ Manitoba Agriculture and Food, Winnipeg, Manitoba, Canada

**Keywords:** surveillance, diagnostics, wicking assay, WNV, corvids

## Abstract

The utility of the VecTest antigen-capture assay to detect West Nile virus (WNV) in field-collected dead corvids was evaluated in Manitoba and Ontario, Canada, in 2001 and 2002. Swabs were taken from the oropharynx, cloaca, or both of 109 American Crows, 31 Blue Jays, 6 Common Ravens, and 4 Black-billed Magpies from Manitoba, and 255 American Crows and 28 Blue Jays from Ontario. The sensitivity and specificity of the antigen-capture assay were greatest for samples from American Crows; oropharyngeal swabs were more sensitive than cloacal swabs, and interlaboratory variation in the results was minimal. The sensitivity and specificity of the VecTest using oropharyngeal swabs from crows were 83.9% and 93.6%, respectively, for Manitoba samples and 83.3% and 95.8%, respectively, for Ontario birds. The VecTest antigen-capture assay on oropharyngeal secretions from crows is a reliable and rapid diagnostic test that appears suitable for incorporation into a WNV surveillance program.

Since 2000, surveillance for West Nile virus (WNV) in dead corvids has been the cornerstone of the early warning system for this virus in the Canadian public health system. During 2001 and 2002, WNV was detected in avian tissues by using real-time TaqMan reverse transcription–polymerase chain reaction (RT-PCR), as described by Lanciotti et al. (*1*). Birds collected by local or provincial authorities were sent to regional centers for tissue collection. Tissue samples were shipped daily, often thousands of kilometers, to the National Microbiology Laboratory in Winnipeg, Manitoba, for final diagnosis (by RT-PCR). Though the sensitivity and specificity of TaqMan RT-PCR assay are excellent, the centralization of testing and the multiple steps necessary to extract bird tissues from carcasses, purify RNA, and screen samples for WNV genome presented major logistical, biosafety, reporting, and financial challenges. Komar et al. (*2*,*3*) demonstrated that WNV is present in high titer in oral and cloacal cavities of experimentally infected birds. Recently, a rapid antigen-capture wicking assay (VecTest, Medical Analysis Systems, Camarillo, CA) has become commercially available (*4*,*5*). Its use for screening swabs from the cloacal or oral cavities of dead birds has yet to be evaluated under field conditions.

The ultimate goal of this study was to determine whether the VecTest assay could serve as a suitable alternative testing procedure for WNV dead bird surveillance. This goal was achieved by quantifying the sensitivity and specificity of this antigen-capture assay to detect WNV in corvids collected as part of routine dead bird surveillance programs in Manitoba and Ontario. The effect of storage conditions and duration of storage of swabs in grinding solution on the sensitivity of the assay and viability of virus was also assessed.

## Materials and Methods

Corvids collected as part of the WNV dead bird surveillance programs in Manitoba and Ontario, Canada, in 2001 and 2002 were used. Laboratories in each province received dead birds, collected oropharyngeal or cloacal swabs or tissues, performed the antigen-capture assay, and shipped tissues or swabs to the National Microbiology Laboratory for confirmatory testing. In both laboratories, only birds lacking signs of obvious decay or decomposition were included for testing. Birds typically were shipped to the laboratories via courier. During submission, most animals were held within insulated coolers containing freezer packs. If specimens could not be shipped on the date of collection, most were held at 4°C. On rare occasions, birds were frozen at –20°C by submitters before shipment or by the diagnostic laboratories before testing. However, most specimens were held at approximately 4°C for 1 to 4 days before processing.

### Ontario/Nunavut Canadian Cooperative Wildlife Health Center

Thirty-one American Crows (*Corvus brachyrhynchos*) and 28 Blue Jays (*Cyanocitta cristata*), collected from June to December in 2001 and held at –20°C were tested in March and April 2002. Swabs were taken from the oropharyngeal cavity and cloaca of each bird. In 2002, testing was restricted to crows (n = 222) from which only oropharyngeal swabs were collected and tested by VecTest. Polyester swabs (VWR CanLab, Mississauga, Ontario) were applied to the oropharyngeal cavity and the cloaca. Swabs were placed in microcentrifuge tubes containing 1 mL of BA-1 diluent (similar to that described by Nasci et al. [*4*]). The antigen-capture assay was carried out according to the manufacturer’s instructions except that the aliquot of swab eluate was 125 μL, not 250 μL. To confirm the infection status of birds, approximately 25-mg pieces of brain and kidney from each bird were pooled in microcentrifuge tubes and stored at –80°C before shipment to the National Microbiology Laboratory. At that laboratory, 1 mL of BA-1 diluent and a tungsten bead (QIAGEN, Inc. [Canada], Mississauga, Ontario) were added to each tube, and tissues were homogenized on a Retsech Mixer mill (QIAGEN). After centrifugation, RNA was extracted from 150- to 200-μL aliquots of supernatant.

### Veterinary Services Branch, Winnipeg, Manitoba

Beginning in mid-August, 2002, a total of 109 American Crows, 31 Blue Jays, 6 Common Ravens (*Corvus corax)*, and 4 Black-billed Magpies (*Pica pica*) were examined. Two cloacal swabs (one each from a Common Raven and an American Crow) and one oropharyngeal swab from a Blue Jay were not taken; hence swabs from both sites were available for a total of 147 birds. Specimens were collected by the procedures described above. However, after the 125-μL aliquots of swab eluate were removed, the remainder of the sample was frozen at –80°C and shipped to the National Microbiology Laboratory for confirmatory RT-PCR testing. Tissues were removed and tested for WNV by using RT-PCR on 42 (29 crows, 9 Blue Jays, 3 Common Ravens, and 1 magpie) of the 150 birds.

In both laboratories, swabs were tested for WNV with the VecTest assay (WNV/St. Louis encephalitis antigen panel), as described by Nasci et al. (*4*), with the following modifications. Swabs were incubated at 37°C for 30 min and shaken intermittently; if debris was present, tubes were centrifuged for 5 min at 4,000 rpm. Aliquots of 125 μL of the manufacturer’s grinding solution and of each swab sample were mixed in a microcentrifuge tube. This mixture was centrifuged by using a Vortex mixer (VWR International, Mississauga, Ontario), and test strips were inserted into the supernatant fluid for 15 min, then removed and examined. During this study, two and five different persons from the Manitoba and Ontario laboratories, respectively, interpreted or read the test strips. As illustrated by Nasci et al. (*4*), presence of WNV was confirmed by the formation of a reddish purple line on the test strip, corresponding to the WNV-positive location and the development of a test control line.

Real-time TaqMan RT-PCR assays to detect WN viral RNA, as described by Lanciotti et al. (*1*), were conducted on all swabs and representative tissue samples from Manitoba and all tissue samples from Ontario. Briefly, RNA was extracted from 150 μL to 200 μL of swab eluates or tissue homogenates by using QIAamp viral RNA kits (QIAGEN). A final volume of 70 μL of eluted RNA was stored at –80°C until used. Five microliters of RNA was combined with the appropriate primers and probes in buffer with the TaqMan RT-PCR ready-mix kit (PE Applied Biosystems, Foster City, CA). Samples were subjected to 40 amplification cycles in an ABI Prism 7700 Sequence Detection System instrument (PE Applied Biosystems), according to the manufacturer’s protocol for TaqMan RT-PCR cycling conditions. All samples were screened with primers and probe specific to the 3′ NTR, and positive extracts were confirmed by reamplification with the ENV primers and probe (*1*). Swabs or tissues were considered positive only if positive results were obtained with both primer and probe sets. Sensitivity and specificity of the antigen-capture assay applied to oropharyngeal and cloacal swabs from corvids were calculated by using the results of TaqMan RT-PCR assays on swab or tissue samples as the “true” outcome (*6*).

To establish how storage conditions and duration of storage affected the sensitivity of the antigen-capture assay, cultures of Egypt 101 strain of WNV (approximately 4 x10^7^ PFU/mL) were serially diluted to 4 X 10^4^ PFU/mL in BA-1 diluent. Polyester swabs were then dipped into each virus solution, placed into 1 mL of manufacturer’s grinding solution, and held at –20°C, 4°C, or room temperature (approximately 18°C). The test strips were applied to aliquots of each solution 0, 3, 5, 7, 10, and 14 days later, as described previously. To assess virus viability in grinding solution, 50 μL of each tested grinding solution and virus dilution was placed into 450 μL of BA-1 diluent, and aliquots of 200 μL of this mixture were added to Vero cells reared on standard nutrient medium (i.e., Dulbecco’s modified Eagle medium and 10% fetal bovine serum [FBS]) at 37°C and 5% CO_2_. After a 1-h adsorption, 2 mL of nutrient medium (same as above but 5% FBS) was added to tissue culture wells, which were incubated as above for up to 7 days. Positive (WNV in BA-1 as above) and negative (media only) controls were maintained under the same conditions, and cells in culture were observed daily for cytopathic effect (CPE). Cells from positive controls and the highest concentration of virus used (i.e., 4 X 10^7^ PFU/mL) in grinding solution for each temperature and sampling date were acetone fixed, exposed to WNV-specific antibodies (produced in rabbits and supplied by H. Weingartl, Canadian Food Inspection Agency, Winnipeg, Manitoba) and conjugated goat anti-rabbit immunoglobulin (Ig) G (Kirkegaard & Perry, Gaithersburg, MD) and assessed for fluorescence under a UV microscope.

## Results and Discussion

The sensitivity and specificity of the VecTest assay for detecting WNV in oropharyngeal and cloacal swabs are presented in the Table. Data from Ontario reflect the sensitivity and specificity of this antigen-capture assay by using swab eluates in relation to the infection status (TaqMan RT-PCR on kidney and brain) of corvids. In contrast, with the exception of 42 birds in which tissue samples were used to establish WNV infection status, the Manitoba results evaluate the sensitivity and specificity of the antigen-capture assay by using swab eluates in relation to the detection of virus in the same sample by TaqMan RT-PCR. Hence, the results obtained in the two laboratories are not directly comparable.

Data from the Ontario birds from 2001 need to be interpreted with caution since sample size was small and the birds were frozen for up to 10 months. However, cloacal swabs appeared to be less sensitive than oropharyngeal swabs in detecting an infected bird. The sensitivity and specificity of the antigen-capture assay, when oropharyngeal swabs from crows collected in 2002 were used, were 83.3% and 95.8%, respectively, indicating that this assay is acceptable for use as a field detection test in a WNV surveillance program. The advantages of this assay compensate for lower sensitivity only in areas where infected crows are relatively common.

The Manitoba data also indicate that this antigen-capture assay is sufficiently sensitive and specific to detect WNV in oropharyngeal and cloacal swabs from crows when compared to RT-PCR results. Sixty-two of 109 crows were positive by RT-PCR on either oropharyngeal or cloacal swab, although the number of birds that would have been positive using tissues was not determined. All but three birds positive on oropharyngeal swabs were also positive on cloacal swabs. Small sample sizes and the relatively small number of positive samples on RT-PCR make evaluation of the utility of this antigen-capture assay on Blue Jays, Common Ravens, and magpies difficult. In general, the test’s specificity seems superior to its sensitivity (Table). Further evaluation is necessary before there can be confidence in this assay as a means of detecting WNV in these species.

**Table:** Sensitivity and specificity of the VecTest assay to detect West Nile virus (WNV) in oropharyngeal and cloacal swabs collected from corvids in Ontario and Manitoba

Species tested (y)	N	Oropharyngeal swabs		Cloacal swabs	
		Sensitivity (%)	Specificity (%)	Sensitivity (%)	Specificity (%)
Ontario					
American Crows (2001)	33	92.8	79	58.3	94.7
Blue Jays (2001)	28	60	100	40	100
American Crows (2002)	222	83.3	95.8	ND^a^	ND
Manitoba					
American Crows	109^b^	83.9	93.6	83.1	97.9
Blue Jays	31^c^	71.4	100	57.1	100
Common Ravens	6^b^	100	100	100	0
Black-billed Magpies	4	0	100	66.7	100

^a^ND, not done. ^b^Cloacal swabs were not available from one bird in each of these groups of birds. ^c^An oropharyngeal swab was not taken from one of the Blue Jays.

In Manitoba, the VecTest assay was not evaluated against RT-PCR on tissue for all birds, and thus the sensitivity and specificity cannot be related to the true infection status of each bird. However, indirect evidence suggests that the Manitoba antigen-capture results approximate those that would have be obtained had RT-PCR on tissues been used in the comparison. On the 29 Manitoba crows in which tissues were used to establish infection status by RT-PCR, the sensitivity and specificity (i.e., 83.3% and 94.1%, respectively) of the antigen-capture assay on oropharyngeal swabs were comparable to those from Ontario. Likewise, the data from Manitoba are similar to the data on sensitivity and specificity of the antigen-capture assay on swab eluates from crows in Ontario and compatible with the high prevalence or titers of virus in oropharyngeal or cloacal samples described by Komar et al. (*2*,*3*).

The “false-negative” VecTest samples had higher mean cycle threshold (CT) values (i.e., CT values are a measure of the overall virus titer in samples; higher CT values indicate lower viral loads) for both primer and probe sets (i.e., 26.1 ∀± 4.5, generic; ± 26.0 ∀± 4.9, envelope) compared to bona fide positives (i.e., 21.0∀ ± 3.4, generic; 19.7∀ ± 3.7, envelope). This observation suggests that the viral titers in the false-negative samples were below the threshold for consistent detection when the antigen-capture format was used. Similar but lower levels of sensitivity have been reported for this antigen-capture assay when it has been used to detect WNV in field-collected mosquitoes (*4*).

Regardless of the storage conditions of swab samples, VecTest strips always detected WNV at viral concentrations >4 X 10^6^ PFU/mL but never at titers of #4 x 10^4^ PFU/mL. The VecTest assay detected WNV at 4 X 10^5^ PFU/mL when the grinding solutions were held at any of the three temperatures for up to 7 days; thereafter WNV was only consistently detected at 4 X 10^5^ PFU/mL when solutions were held at –20°C. Grinding solution appears to inactivate WNV. None of the tissue cultures inoculated with grinding solution containing WNV had evidence of CPE or positive indirect fluorescent-antibody assay results, whereas all of the positive controls did. Based on these results, swabs can be placed in grinding solution for up to 7 days before the test strips are applied and held from –20°C to 18°C, without loss of sensitivity. As noted by Nasci et al. (*4*), once in the grinding solution, the titer of WNV is substantially reduced, as would be any potential biological hazard associated with handling or shipping the swab samples.

In noncorvid species the VecTest assay also has low sensitivity when compared with immunohistochemistry to detect viral antigen in fixed tissue and RT-PCR on frozen tissue. The sensitivity of this antigen-capture assay was 46.7% for oropharyngeal swabs taken from 27 different raptors (of various species) in Ontario during 2002 (G.D. Campbell, Ontario Veterinary College, ON, pers. comm.). This obviates the use of this assay as a reliable screening test for WNV infection in rehabilitation centers, veterinary clinics, zoos, animal disease diagnostic laboratories, and other settings where potential exposure to a biocontainment level 3 agent is of concern.

The advantages of this antigen-capture assay over conventional molecular-based diagnostic procedures such as real-time TaqMan RT-PCR include simplicity of procedures, no requirement for expensive and technically demanding instruments, and much shorter turn-around times for testing (i.e., results are available in 15 min). In addition, testing swabs rather than tissues eliminates the need to dissect submitted birds, thus decreasing the processing time and cost of testing and the risk for lacerations to laboratory workers (*7*).

The greatest limitation to the use of this system in WNV surveillance programs is the potential loss of information on early season WNV activity. This loss could result from the lower sensitivity of the antigen-capture assay compared to the real-time RT-PCR TaqMan assay. However, the loss of temporal sensitivity likely would be offset somewhat by the rapid turn-around times possible with the antigen-capture format. In addition, in our experience, the VecTest assay does not appear to work as effectively in noncorvid (or all corvid) species. Thus, its usefulness would be greatly diminished in jurisdictions that test all avian species as part of their WNV surveillance programs. The poorer performance of the VecTest assay in noncorvids may be related to species-specific differences (and variability) in virus titers in excretions and secretions at the time of death (*3*). The VecTest assay will likely replace TaqMan RT-PCR assays as the front-line diagnostic test for future WNV dead bird surveillance programs in Canada. When this antigen-capture assay is used, only index cases of WNV infection in dead birds detected in a given jurisdiction such as a health unit or municipality need to be confirmed with RT-PCR assays before public notification of virus activity. This procedure would compensate for the slightly lower specificity of the antigen-capture assay and should result in markedly decreased workloads for laboratories using molecular diagnostic procedures. VecTest positives obtained thereafter need not be confirmed by an alternative assay since the impact of false positives in a jurisdiction with previously confirmed virus activity should be negligible.
